# Factors Predicting the Adherence to the Therapy of Italian IBD Patients

**DOI:** 10.1155/2017/6719345

**Published:** 2017-08-07

**Authors:** Cristina Bucci, Fabiana Zingone, Stella Tammaro, Paola Iovino, Antonella Santonicola, Carolina Ciacci

**Affiliations:** Department of Medicine and Surgery, Scuola Medica Salernitana, AOU San Giovanni di Dio and Ruggi d'Aragona, IBD Center at the University of Salerno, Salerno, Italy

## Abstract

**Background:**

Inflammatory bowel diseases (IBD) are chronic gastrointestinal disorders influencing many aspects of the patient's life and accounting for substantial social costs. They require long-term therapies and regular contact with the clinic of reference. Our aim is to investigate therapy adherence and identify predictors of adherence.

**Methods:**

151 patients were recruited in IBD clinic at the University of Salerno filled in the modified Morisky Medication Adherence Scale, a standardized questionnaire provided during the visit.

**Results:**

Overall, 71.5% of the patients report to take all medications regularly. According to the scale, our population showed a 62.5% medium-high adherence to oral 5-ASA, a 72% medium–high adherence to immunomodulators, a 60% medium–high adherence to oral steroids, and 94.9% adherence to biologics. Younger patients tend to be less compliant to the therapy. The main reasons for the low adherence to therapy were the “hassle of sticking to the medication plan” and “their feeling better.” *Conclusion*. In Italy, where the healthcare system covers most of the expenses for IBD therapy, almost 30% of IBD patients report low compliance to therapy. Healthcare givers should improve the knowledge regarding the disease and favor the development of combined drugs that would simplify the daily medication plan.

## 1. Introduction

Inflammatory bowel diseases (IBD) are chronic medical conditions that require long-term therapies [1] and regular contact with the clinic of [2]. IBD mostly begins in young people and generally goes on with alternate courses of remission and flare which could lead to complications such as surgery and hospitalization. For these reasons, adherence to medical therapies is essential to maintain the disease in a remission phase and avoid negative consequences. Adherence has been defined as the “extent to which a person's behavior (taking medications, executing lifestyle changes, undergoing tests, attending scheduled appointments) corresponds with the recommendations from a health care provider” [3, 4]. Currently, five types of drugs are useful in IBD, and patients will likely have to take one or all of them at a certain moment of their life: steroids, occasionally antibiotics, immune modifiers such as azathioprine and 6-mercaptopurine, aminosalicylates, and/or biologic therapy. The data regarding IBD patients show that nonadherence to therapy is associated with inactive disease, 5-aminosalicylic acid (5-ASA) oral medications, younger age, longer intervals between outpatient clinic visits (≥3 mo), and limited knowledge of the prescribed medication [5, 6]. Nonadherent patients had a significantly greater risk of relapse of IBD, reduced quality of life, and increased costs of care [7, 8].

Just like for other diseases, the strategies to improve therapy adherence in IBD include regimen simplification, decrease of extra costs, and the cooperation of health professionals. Little is known about the effects of technology-mediated interventions in IBDs, such as reminders via cellular phone applications, email alerts, and/or telemedicine healthcare, although their effectiveness may enhance patient-specific approaches to improve adherence and disease control [9–11].

Different tools to assess therapy adherence levels were tested in clinical and research settings (e.g., circulating drug or metabolites levels, pharmacy refill, patient questionnaires, interviews, and diaries) and, among the others, self-report subjective measures appeared to be more reliable than the objective ones [12]. One of the questionnaires that have been used to measure therapy adherence is the modified Morisky Medication Adherence Scale (MMAS-8), a self-administered questionnaire, initially validated in patients on antihypertensive medications and then tested also in IBD [13–17]. In 2011, Trindade et al. validated this questionnaire in IBD, concluding that it could identify patients with poor adherence better than physicians and thus could serve as a useful tool in practice [18].

Data regarding IBD therapy adherence from Italy are missing, except for two studies that, using nonvalidated questionnaires, showed that nonadherence to therapy was more frequent in patients younger than 40 years [19], females, followed-up by general practitioners [20], regardless of socio-demographic characteristics or type of IBD.

The aim of our study was to investigate IBD therapy adherence in a series of Italian patients and identify predictors of adherence.

## 2. Methods

Consecutive patients were recruited in IBD outpatient clinic at the University of Salerno, Italy, from January 2016 to July 2016, with a diagnosis of IBD according to the usual clinical, endoscopic, radiologic, or histopathologic criteria. Exclusion criteria were IBD diagnosis < 2 months, patients younger than 18 or older than 75 years, patients reported to feature psychotropic drug abuse or alcoholism, and pregnant women. At the end of the visit, the physician left the room. The patient were asked to fill in, by means of a research dedicated device and the help of a medical student with access to the clinical records, an online anonymous questionnaire containing clinical data such as age (grouped in <30, 31–50, and >50 years), sex, diagnosis (CD or UC), length of illness (<1 year, 1–5 years, or >5 years from diagnosis), disease activity (patients' judgment) on the day of the visit, and medications for IBD or other concomitant medications. These variables were chosen since they may be associated with therapy adherence. Patients were asked to fill in the modified Morisky Medication Adherence Scale (MMAS-8). The MMAS-8 is an 8-question survey with 7 questions having a binary answer (yes/no) formulated to avoid the “yes-saying bias,” while the last question is a five-point Likert response that explores a specific medication-taking behavior. The MMAS-8 is based on the idea that low adherence could occur because of many reasons, such as failure to remember to take a medication or pharmacy refill and problems with the complexity of medical regimens. As patients could be on multiple IBD medications, data were separately collected on each drug (oral 5-ASA, steroids, immunomodulators, and biologics). For intravenous biologics (e.g., infliximab), the questions meant to explore if the patient had ever forgotten to take medication on the due date was reformulated asking if they had ever forgotten to attend the hospital infusion. As described by Trindade et al., a score of 8 indicates high adherence, 6 to 8 medium adherence, and <6 low adherence. The results were analyzed combining medium and high adherence, as previously validated [18]. Morisky directly provided the validated Italian translation and the survey scoring.

Apart from the MMAS-8, patients were also asked to grade the importance of taking all medication with respect to their disease through a five-point Likert scale (from 0 “not very important” to 4 “very important”) and to self-report which is in general the most frequently missed IBD drug (oral 5-ASA, immunomodulators, biologics, or steroids).

The study was approved by the Ethics Committee of the University of Salerno. All patients signed the informed consent.

## 3. Statistical Analysis

Categorical variables were expressed as frequency, and differences in frequencies among groups were calculated using the *χ*^2^ test. Firstly, univariate logistic regression models were used to assess whether all possible confounders (sex, age groups, diagnosis, disease activity, length of disease, and concomitant medications) were separately related to the therapy adherence. Secondly, multivariate full models were built that included all the confounders taken into account. All tests were two-tailed with the significance level set at *p* < 0.05. STATA 12 software was used to analyze the data.

## 4. Results

During the study period, 151 IBD patients were consecutively recruited (53.6% males).  Table 1 summarizes the main characteristics of our study population: 38.4% were under 30 years old and 23.8% were over 50 years old, 42.4% had CD, 58.9% were in remission at the time of the study, and 49% had been diagnosed IBD more than 5 years before. Roughly one-third of our population (27.8%) were taking medications other than those for IBD.

Apart from one dissenter, all eligible patients agreed to answer the questions in the survey. Overall, on a 5-point Likert scale, patients regarded taking their medication as “very important” (66.9%) or “important” (19.9%) in the management of their disease. Accordingly, considering the last 6 months, 71.5% self-reported to have taken all medication regularly, while 18.5% reported to have forgotten oral 5-ASA most frequently, and 1.3% oral low-availability steroids such as budesonide. Only 3 patients admitted forgetting to take injectable anti-TNF agents (2 patients) or to show up for the infusive anti-TNF agent (1 patient).

According to the MMAS-8 scale, our population resulted to be therapeutically adherent, showing a 62.5% medium–high adherence to oral 5-ASA, a 72% medium–high adherence to immunomodulators, a 60% medium–high adherence to oral steroids, and 94.9% adherence to biologics (Table 2). When anti-TNF agents were subdivided between injectable (16 patients) and intravenous (23 patients), biologics' results showed that all but one patient in both groups self-reported to be highly adherent (Table 2). Interestingly, the clinical and demographic characteristics of these 2 nonadherent patients were similar: both were women, younger than 30, with Crohn's disease for more than 5 years, and on concomitant 5-ASA therapy, to which they reported to be highly adherent.

Oral 5-ASA was the most commonly taken medication in our population (128/151 patients), so we looked at all factors that may influence the therapeutic adherence. As shown in Table 3, in the univariate analyses, the factors were as follows: being 31–50 years old [OR 2.41 (95% CI 1.04–5.54)] or over 50 [OR 7.45 (95% CI 2.46–22.56)], disease duration > 5 years [OR 3.61 (95% CI 1.11–11.74)], and taking concomitant non-IBD medications [OR 3.51 (95% CI 1.40–8.82)]. Also, according to our data, having a UC and being on an active flare tends to increase the therapeutic adherence to 5-ASA, but these results were not statistically significant. However, performing a multivariate analysis where all factors were reciprocally adjusted, only being over 50 is clearly associated with a medium–high compliance in our population [OR 5.18 (95% CI 1.47–18.2)] (Table 3).

We also evaluated if taking concomitant “more effective” IBD medications such as biologics or immunomodulators could affect the adherence to 5-ASA or vice versa, showing that patients on 5-ASA and immunomodulators or 5-ASA and biologics had the same adherence as those taking 5-ASA only (data not shown).  Table 4 summarizes the reasons why some patients stopped taking medications, according to the questions in the MMAS-8 survey. The most frequent answer was that “they felt hassled about sticking to the therapy plan.”

## 5. Discussion

IBD treatment is an issue in the clinical practice. It may require taking different medications at the same time and with different regimens, as well as lifestyle and dietary modifications, despite the fact that the results are often nonreadily appreciable both by patients and by clinicians. Just like other chronic diseases, IBD therapeutic adherence underlies a close doctor-patient relationship and personal and familiar surroundings that could support the patients' choice [21–23].

The present study indicates that, overall, 71.5% of our patients self-reported to take all medication regularly, with 5-ASA and oral steroids being the most frequently forgotten medications. According to the MMAS-8 scale, the best-reported adherence was to biologics (94.9%), with no difference between injectable or intravenous anti-TNF, and to immune-modulators (72%), while the worst adherence was reported to 5-ASA (62.5%) and oral steroids (60%). The prescription of different IBD medications at the same time does not seem to affect adherence, and no associations were noticed regarding gender, type of disease, length, or disease activity.

Oral 5-ASA was the most commonly taken medication (128/151 patients), and the factor associated with high adherence to 5-ASA was being older than 50, but no association was noticed with concomitant coprescriptions or length of disease.

Our results are in line with previous studies that have shown that nonadherence to medication prevalently ranges from 35% to 72% [24–29] and is even higher than expected for biologics (82.6%) [30]. A UK study revealed that nearly 15% of patients fail to even redeem prescriptions at the pharmacy [31]. In 2008, D'Incà et al. used a nonstandardized anonymous 24-item questionnaire for 485 outpatients attending a tertiary referral center in Padua. Results showed that overall therapeutic adherence was 61%, with nonadherence being significantly associated only with patients under 40 years old (43% versus 34%, *p* = 0.041) [19]. In 2014, Zelante et al. found that the factor independently associated with medical adherence in 559 outpatients followed by both general practitioners and gastroenterologists was age (OR = 2.039) and when followed up by a gastroenterologist (OR = 3.025); no difference was found in educational status or type of IBD [20].

The strengths of the present work are firstly that, to our knowledge, this is the first Italian study dealing through a validated tool with the therapeutic adherence to IBD medications in a homogeneous cohort of IBD patients. In fact, all patients but one agreed to participate in the survey; gender, age, and disease activity and duration were equally distributed, and the same tertiary center followed all.

Secondly, and more importantly, all patients were in the same economic conditions as regards to the national health system, which covered all the IBD patients of our cohort for direct and indirect costs related to the disease. It is estimated that, among the other expenses (outpatient services, diagnostic procedures, hospitalizations, etc.), the pharmacy utilization is the major direct cost driver of total health-plan paid costs for Crohn's disease in the USA, accounting for 35% of the total amount (about $7000 per patient/year) [32]. Similar data are available for Europe [33]. However, one can speculate that from the patients' perspective, there are differences in the access to healthcare, for example, the private practice and healthcare influencing a better patients' compliance. This is not the case in our population, as the Italian IBD patients are in the best conditions to adhere to the therapy as they do not bear most of the economic burden of the disease and receive a high-quality assistance. As a matter of fact, Italian IBD patients report to be overall satisfied with the quality of the healthcare assured [34].

Some limitations of this study need to be addressed as well. Firstly, although MMAS-8 tool was modified to measure the adherence to anti-TNF agents, some questions may not apply to nonoral medications. Secondly, when divided according to medications, the sample size of our study becomes underpowered to detect all factors that could influence adherence to biologics or immunomodulators. For this reason, we chose to perform the main analysis using the medication taken by most of our population. Thirdly, our cohort came from the same geographic area (Salerno city and its province) and this may not reflect the behaviour of the whole Italian population; thus, a multicentre Italian study would be advisable in the future. Fourth, the activity of disease was self-defined by the patients and not by the physicians because we tried to minimize the influence of physicians on the patients' answers and also because in IBD clinical scales (such as CDAI or the complete MAYO score) are troublesome and do not always reflect the patient's perception of his/her health status.

The most frequent reason for stopping taking medication was that patients did not feel like sticking to the therapy plan. Therefore, the development of long-acting combined drugs may overcome the difficulty of taking several medications at different times of the day.

Most importantly, our results underline that young people are at greater risk of being noncompliant to therapy and indicate that healthcare givers should be more careful to improve the knowledge of IBD and the importance of treatment adherence in younger patients.

## Figures and Tables

**Table 1:** Study population characteristics (151 patients).

Variables	*N* (%)
Males	81 (53.6)
*Age groups*	
<30 years	58 (38.4)
31–50 years	57 (37.7)
>50 years	36 (23.8)
*Type of disease*	
CD	64 (42.4)
UC	87 (57.6)
*Disease activity*	
Remission	82 (58.9)
Active	62 (41.1)
*Time from diagnosis*	
<1 year	18 (11.9)
1–5 years	59 (39.1)
>5 years	74 (49)
Concomitant drugs	42 (27.8)

CD = Crohn's disease; UC = ulcerative colitis.

**Table 2:** Type of drugs and reported compliance according to MMAS-8.

Drug class	Number of users	Grade compliance	*N* (%)
Oral 5-ASA	128	Low	48 (37.5)
		Medium–high	80 (62.5)
Immunomodulators	25	Low	7 (28)
		Medium–high	18 (72)
Oral steroids	15	Low	6 (40)
		Medium–high	9 (60)
Injectable biologics	16	Low	1 (6.3)
		Medium–high	15 (93.7)
Intravenous biologics	23	Low	1 (4.4)
		Medium–high	22 (95.6)

Low adherence (<6 points); medium–high adherence (6.1 to 8); *N* = numbers; MMAS-8 = modified Morisky Adherence Scale; 5-ASA = 5-aminosalicylic acid; the use of MMAS is protected by US copyright laws. Permission for use is required. Licensure agreement is available from Donald E. Morisky, ScD, ScM, MSPH, Professor, Department of Community Health Sciences, UCLA School of Public Health, 650 Charles E. Young Drive South, Los Angeles, California 90095, USA.

**Table 3:** Oral 5-ASA compliance (high/moderate versus low) according to different confounders.

	*N*	OR (95% CI)	aOR (95% CI)^∗^
*Gender*			
Males	72	1	1
Females	56	0.76 (0.37–1.56)	0.77 (0.35–1.71)
*Age groups*			
<30 years	48	1	1
31–50 years	46	2.41 (1.04-5.54)	1.79 (0.69–4.60)
>50 years	34	7.45 (2.46–22.56)	5.18 (1.47–18.2)
*Type of disease*			
CD	50	1	1
UC	78	1.37 (0.66–2.84)	1.64 (0.70–3.83)
*Disease activity*			
Remission	77	1	1
Active	51	1.35 (0.64–2.82)	1.62 (0.70–3.74)
*Time from diagnosis*			
<1 year	15	1	1
1–5 years	55	2.25 (0.70–7.21)	2.20 (0.61–7.95)
>5 years	58	3.61 (1.11–11.74)	2.64 (0.71–9.81)
*Concomitant drugs*			
No	91	1	1
Yes	37	3.51 (1.40–8.82)	2.06 (0.73–5.83)

^∗^Adjusted for sex, age groups, type of disease, disease activity, time from diagnosis, and concomitant drugs when nonstratified for. CD = Crohn's disease; UC = ulcerative colitis.

**Table 4:** Prevalence of reported reasons for stopping taking medications in IBD patients. The questions were repeated for each medication.

Questions taken from MMAS-8	5-ASA *N* (%)	IMMs *N* (%)	Steroids *N* (%)	Biologics *N* (%)
Have you ever cut back or stopped taking medications without telling your doctor because you felt worse when you took them? (Yes, %)	37 (29)	4 (16)	2 (13.4)	3 (7.7)
When you travel or leave home, do you sometimes forget to bring along medications? (Yes, %)	16 (12.5)	3 (12)	1 (6.7)	2 (5.1)
When you feel like your IBD symptoms are under control, do you sometimes stop taking medication or showing up for clinical appointment? (Yes, %)	40 (31.3)	4 (16)	3 (20)	2 (5.1)
Taking the medication with the right schedule is a real inconvenience for some people. Do you ever feel hassled about sticking to the therapy plan? (Yes, %)	63 (49.2)	12 (48)	7 (46.7)	17 (43.6)

MMAS-8 = modified Morisky Adherence Scale; 5-ASA = 5-aminosalicylic acid; IMMs = immunomodulators.
